# Apoptosis Versus Survival: When in Doubt, Choose Both

**DOI:** 10.1371/journal.pbio.0040408

**Published:** 2006-11-07

**Authors:** Richard Robinson

To function, a protein must fold up after it is synthesized. When it doesn’t, trouble can ensue—the accumulation of unfolded proteins is a common cause of degenerative disorders such as Parkinson disease. When unfolded or misfolded proteins begin to build up, the cell sets in motion a coordinated cascade of events termed the “unfolded protein response” (UPR). In fact, the UPR comprises two distinct pathways leading to opposite fates: down one path lies adaptation and continued life for the cell, while down the other lies the programmed cell death known as apoptosis.

It has been known for some time that the choice of pathway depends in part on the amount of unfolded protein: small amounts lead to adaptation and large amounts to death. But how does the cell select between these two paths? In a new study, Thomas Rutkowski, Randal Kaufman, and colleagues show that even when unfolded protein levels are low, both paths are activated. But the signals that lead to apoptosis are short-lived, and in the absence of high amounts of unfolded protein, they cannot trigger further events in the apoptotic chain before they are degraded.

The authors began by treating cells in culture with either one of two chemicals that disrupt protein folding, activating the UPR. At high chemical levels, the cells died. But at low levels, the cells adapted to the stress, continuing to proliferate, largely avoiding apoptosis, and reducing the level of unfolded protein over time. In addition, once the cells had undergone adaptation, they were more resistant to further stress, either from the original stressor chemical, or from the other one of the pair.

The UPR begins when a protein called BiP, normally bound to three other proteins, instead binds increasingly to accumulating unfolded protein. Without BiP, these three proteins—called PERK, IRE1, and ATF6—become activated, which is the crucial trigger for both the adaptive and terminal responses. The authors showed that both high and low chemical concentrations activated the three proteins, indicating that the difference in outcome was not due to a failure to start down the apoptotic pathway. However, another protein was differentially affected by chemical concentration. CHOP is a pro-apoptotic protein further down the cell-death pathway. Both high and low chemical concentrations stimulated CHOP production, but when the chemicals were at low concentration, the increase in CHOP didn’t last, and the cells survived. BiP was also increased by the two chemicals, but unlike CHOP, its up-regulation persisted at low concentrations.

This difference between BiP and CHOP was not due to a difference in the amount of mRNA produced from the two genes. Instead, the authors showed, the CHOP mRNA was less stable, degrading within hours, while the BiP mRNA persisted. Thus, BiP proteins continued to be produced long after CHOP production had ceased. Similarly, the BiP protein was more stable than the CHOP protein. The excess of BiP allowed the three protein triggers—PERK, IRE1, and ATF6—to be re-bound and decommissioned, while the cell increased its production of other proteins to reduce the level of unfolded proteins that began the entire process. Only when the stressing chemicals were at high concentration did enough CHOP protein last long enough to tip the balance toward apoptosis rather than adaptation.

**Figure:**
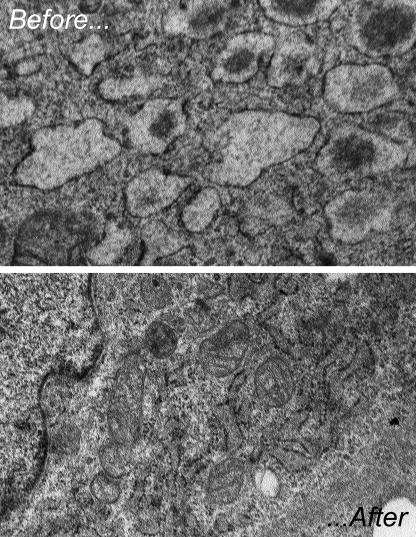
Electron microscopic images of the endoplasmic reticulum in mouse embryonic fibroblasts that are either newly exposed (top) or adapted (bottom) to stress.

Finally, the authors showed that the same prolonged adaptive response can occur not only when the stress arises from chemical insult but also from a genetic defect in the protein quality control machinery. Challenged with chemical endoplasmic reticulum stress, such cells were relatively resistant to damage, demonstrating a pre-existing adaptation due to the chronic low-level stress as a consequence of the genetic mutation.

These results highlight a versatile strategy for choosing in the face of uncertainty: when in doubt, choose both. When stressed, the cell initiates both survival and death pathways. The system is weighted toward survival, but if the situation continues to be dire and apoptosis becomes the preferred result, the cell is primed for its final act. Further exploration of this system is likely to reveal more about programmed death and its alternative, and how the cell chooses between them.

